# Navigating Diagnostic and Treatment Challenges of Pulmonary Hypertension in Infants with Bronchopulmonary Dysplasia

**DOI:** 10.3390/jcm13123417

**Published:** 2024-06-11

**Authors:** Nidhy P. Varghese, Gabriel Altit, Megan M. Gubichuk, Roopa Siddaiah

**Affiliations:** 1 Department of Pediatrics, Division of Pulmonology, Baylor College of Medicine and Texas Children’s Hospital, 6701 Fannin St., Ste 1040, Houston, TX 77030, USA; 2 Division of Neonatology, Department of Pediatrics, Montreal Children’s Hospital, McGill University, Montreal, QC H4A 3J1, Canada; gabriel.altit@mcgill.ca; 3 Division of Pulmonary and Sleep Medicine, Children’s Mercy Hospital, Kansas City, MO 64108, USA; mgubichuk@cmh.edu; 4 Department of Pediatrics, Penn State Health Children’s Hospital, Hershey, PA 17033, USA; rsiddaiah@pennstatehealth.psu.edu

**Keywords:** bronchopulmonary dysplasia, pulmonary hypertension, echocardiography, pulmonary vein stenosis, pulmonary vascular disease, prematurity

## Abstract

Advances in perinatal intensive care have significantly enhanced the survival rates of extremely low gestation-al-age neonates but with continued high rates of bronchopulmonary dysplasia (BPD). Nevertheless, as the survival of these infants improves, there is a growing awareness of associated abnormalities in pulmonary vascular development and hemodynamics within the pulmonary circulation. Premature infants, now born as early as 22 weeks, face heightened risks of adverse development in both pulmonary arterial and venous systems. This risk is compounded by parenchymal and airway abnormalities, as well as factors such as inflammation, fibrosis, and adverse growth trajectory. The presence of pulmonary hypertension in bronchopulmonary dysplasia (BPD-PH) has been linked to an increased mortality and substantial morbidities, including a greater susceptibility to later neurodevelopmental challenges. BPD-PH is now recognized to be a spectrum of disease, with a multifactorial pathophysiology. This review discusses the challenges associated with the identification and management of BPD-PH, both of which are important in minimizing further disease progression and improving cardiopulmonary morbidity in the BPD infant.

## 1. Introduction

Bronchopulmonary dysplasia (BPD) is a chronic lung disease primarily affecting premature infants and characterized by an abnormal development of the lungs. While fundamentally representing distorted pulmonary architecture leading to an abnormal function, its clinical definition and severity stratification is often based on the presence and degree of respiratory or oxygen support at 36 weeks postmenstrual age (PMA) in preterm infants born at <32 weeks of estimated gestational age [1,2]. The understanding of this condition has evolved over time, with recognition of shared respiratory, airway, and vascular maldevelopment in its pathogenesis [3]. The development of pulmonary hypertension (PH) has been described as high as 40% in this group and is associated with a significant increase in morbidity and mortality [4]. Therefore, the importance of early identification is crucial and guidelines have now advocated for the screening of PH in those with established or evolving BPD [5,6]. 

Infants with BPD may prompt concern for PH by displaying nonspecific signs and/or symptoms. These may include frequent episodes of hypoxemia, respiratory deterioration events necessitating an escalation of respiratory or oxygen support, challenges in handling, suboptimal growth, requirement for sedation and neuromuscular blockade to facilitate optimal ventilation, slow clinical progress, hepatomegaly, cardiac murmur, episodes of significant hemodynamic deterioration, or shock with acidosis and increased lactate [7]. These concerning elements, particularly when coupled with recognized perinatal risk factors such as maternal preeclampsia, chorioamnionitis, intrauterine growth restriction, prolonged rupture of membranes, oligohydramnios, sepsis, and necrotizing enterocolitis, may raise suspicion that these clinical observations indicate an underlying abnormality in pulmonary vascular development and function [8]. However, BPD infants may be “asymptomatic” of their underlying pulmonary vascular disease considering significant underlying cardiac compensation—hence the recommendation for screening through the aggregation of serial clinical assessments, echocardiography, cardiac catheterization, and adjunct evaluations such as laboratory investigations and dedicated imaging with complimentary modalities.

The diagnosis of BPD-PH represents a negative prognostic factor for mortality in the premature population, especially within the initial two years of life [7]. This disease, which is characterized by elevated pulmonary vascular resistance resulting from increased constriction, muscular vascular thickening, reduced vascular territory, heightened pulmonary reactivity, ventilation–perfusion mismatch, and potentially adverse venous drainage and remodeling [9,10,11], represents a heterogeneous lung disorder influenced by immature repair mechanisms, pulmonary inflammation, and fibrosis. The physiological complexities are further compounded by factors like shunt physiology through persistent systemic-to-pulmonary connections such as patent ductus arteriosus (PDA), which increase pulmonary blood flow, leading to reactive constriction and exposing the pulmonary vasculature to systemic pressures. However, the role of the PDA as a *causal* contributor to BPD-PH remains unclear [12]. Fortunately, ongoing lung growth during this period results in heightened pulmonary vascular capacitance and improved respiratory units; therefore, well-managed cases of BPD-PH often demonstrate improvement [13,14]. Central to this process is an emphasis on optimizing cardiorespiratory status, preventing infections, and prioritizing linear growth. The favorable effect on prognosis underscores the significance of overcoming challenges in detection and confirmation, and highlights the need for prompt initiation of multidisciplinary management to achieve this prognostic effect [15]. Yet herein lies one of the biggest challenges in approaching the BPD infant with PH—how can there be certainty in the diagnosis to guide management?

We present a review of the current literature published on the topic of BPD-PH, focusing on screening recommendations and practices, the use of echocardiography as a diagnostic tool, and report on general, recommended strategies for management of BPD-PH. As bedside clinicians from four different institutions, we also highlight practical challenges in the care of the BPD-PH infant. We provide an algorithm to guide screening, workup, diagnosis, and management, recognizing the limitations inherent to the literature review and reliance on consensus experience where there is a lack of evidence to guide practice.

## 2. Challenge #1: Screening and Confirmation 

### 2.1. Screening for Pulmonary Hypertension

In the first three months of life, pulmonary vascular resistance naturally falls to normal adult levels. PH has been defined as values of mean pulmonary arterial pressure (mPAP) above 20 mmHg, largely based on the adult literature of idiopathic pulmonary arterial hypertension and the associated risk of mortality or lung transplant with this cutoff [16,17]. The normal adult pulmonary artery pressure at rest is typically around 12–16 mmHg for the systolic pressure, 8–12 mmHg for the diastolic pressure, and an mPAP of 9–12 mmHg. While pulmonary artery pressure at birth is elevated due to an increased blood flow into pulmonary vessels postnatally, this pressure gradually drops in the first few days of life to approach these adult levels [18]. 

PH is defined as an abnormally high pulmonary pressure in the pulmonary artery. This condition leads to end-organ damage; in this case, right ventricular (RV) systolic and diastolic failure. The underlying mechanisms to the increased pulmonary pressure in the context of BPD may be related to increased pulmonary vascular resistance, decreased pulmonary vascular capacitance due pulmonary hypoplasia, increased flow to the pulmonary artery by persistent fetal shunt, postcapillary phenomenon (pulmonary vein stenosis, pulmonary venous occlusive disease, left ventricular diastolic failure), or a combination of these elements. Screening practice for BPD-PH is recommended at 36 weeks PMA [6]. However, there is a high variability in this practice. While only 38% respondents from a survey of North American neonatal consultants had implemented a screening program for BPD-PH [15], this program varied in its timing from 28 days after birth to 36 weeks PMA based on a systematic review analysis and lacks consensus [19].

### 2.2. Echocardiography as a Screening and Diagnostic Tool

Echocardiography enables the assessment of cardiac anatomy, underlying cardiac function, and estimation of pulmonary arterial pressure using various metrics (Table 1 and Table 2). This diagnostic modality is noninvasive, radiation-free, and facilitates the estimation of pulmonary arterial pressures under real-life circumstances, eliminating the need for general anesthesia. It also allows for serial follow-up for surveillance, initiation, or titration of therapy. However, there are limitations in this population owing to operator variations, suboptimal imaging windows due to lung disease, tracheostomy presence, critical illness, testing availability, and/or cardiologist familiarity with BPD/BPD-PH echocardiography reading protocols. As per current guidelines [5,20,21], premature infants should undergo echocardiography screening for PH in several specific scenarios. Primarily, screening is recommended at the time of BPD diagnosis, typically around 36 weeks PMA. A corollary to this is the consideration for early screening at postnatal day 7 for the premature infant with a continued need for ventilator support, because evidence of PH at this early stage suggests a high risk for developing BPD at 36 weeks and an early echocardiographic assessment may influence therapeutic decisions [6,22,23]. Lastly, infants requiring sustained significant respiratory support at any age, particularly those with recurrent episodes of hypoxemia, should undergo echocardiography to assess for the presence of PH. These screening scenarios aim to detect and manage PH early in premature infants, allowing for timely interventions and optimized care. It should be noted that screening is recommended in cases of perinatal severe hypoxemic respiratory failure, despite optimal management of underlying lung disease, irrespective of gestational age at birth, to identify developmental lung diseases known to be associated with PH. 

When used for screening, a normal echocardiogram in an infant with BPD will have an estimated RVSP of less than one-third of the systemic pressure, a rounded septal position at peak of systole (round LV in parasternal short axis view indicating higher LV pressure compared to RV pressure), and an absence of RV changes (hypertrophy, dilation, and functional deterioration). “Mild” PH is suspected when the RVSP is more than one-third of the systemic systolic blood pressure at the time of echocardiography [6], with septal flattening in systole, mild RV hypertrophy (RVH), and RV dilatation (which can be quantified by the RV–LV diameter ratio in parasternal short axis; a normal ratio is below 1.0). Some authors have used other cutoffs of TRJ, such as an RV–RA gradient of 33.6 mmHg [12]. The “moderate” PH category is suspected when the RVSP is between half and two-thirds of the systemic pressure. Echocardiographic features include a flat septum or late systolic posterior bowing, moderate RVH or dilatation, and a potential reduction in RV function. “Severe” PH is diagnosed when the RVSP exceeds two-thirds of the systemic pressure. If shunts are present, there is a predominant right-to-left shunt. Pansystolic posterior septal bowing is observed, along with severe RVH, RV dysfunction, and RV dilatation. In the context of a PDA or a VSD, “low velocity” shunting is often noted. These gradations provide a comprehensive framework for assessing the severity of PH and their associated hemodynamic changes. Of note, a straight ventricular septum in diastole usually indicates right ventricular volume overload, often seen in the context of a chronically significant left-to-right shunt at the interatrial level, raising Qp:Qs. This is uncommonly seen in the first few months of life, because the magnitude of the shunt increases over time following the transition to postnatal life and cardiac adaptation to the increased volume load. A straight ventricular septum in diastole may also be seen in the context of increased RV end-diastolic pressure, which may be secondary to RV hypertrophy and altered RV performance. In the setting of chronically increased RV pressure overload with RV adaptation, one may sometimes appreciate adverse septal configuration throughout the cardiac cycle. In this context, flattening of the septum is appreciated in both systole and diastole due to RV systolic and diastolic impairments [57,58].

Other echocardiographic markers [59] may also provide more subtle information on systolic and diastolic performance, such as tissue Doppler imaging, filling velocities, pulmonary vein velocities, deformation by speckle-tracking analysis, and 3D-echocardiographic–derived ejection fraction. RV size may be evaluated according to Z-scores [60] and interpreted for dilation. Assessment of RV function at the initial screening is important because the initial altered ventricular function at PH detection has been associated with an increased mortality [32,50]. Functional measurements can be achieved through assessment of tricuspid annular plane systolic excursion (TAPSE) [35] and fractional area change (FAC). A comprehensive evaluation of the left ventricle is also of importance due to the interventricular interactions. 

Shunting directionality through a post-tricuspid shunt informs on the pressure relationship between the pulmonary and systemic side. In the context of an unrestrictive post-tricuspid shunt, the sPAP pressure is, by definition, close to the systemic pressure due to the equalization of pressure on both sides of the shunting lesion and, therefore, will not be helpful to draw conclusions on underlying vascular resistances. However, a restrictive shunt can give significant information. As such, a right-to-left ventricular shunt informs that the right-sided pressure is higher than the left-sided pressure. Atrial-level shunts reflect the diastolic compliance of the respective ventricles [49] and so a right-to-left shunt suggests impaired diastolic filling pressures of the right ventricle, a condition seen in right ventricular diastolic failure. 

### 2.3. Confirmation of Diagnosis

#### Cardiac Catheterization Is Not Routinely Performed 

While cardiac catheterization is acknowledged as the gold standard for diagnosing PH, there is a lack of universal recommendations advocating for its routine use in infants with BPD [61]. Cardiac catheterization plays a crucial role not only in confirming the diagnosis of precapillary PH (defined as an mPAP greater than 20 mmHg, pulmonary arterial wedge pressure or LV end-diastolic pressure less than or equal to 15 mmHg, and a PVR indexed to body surface area greater than or equal to 3 Woods unit ×m^2^ in age > 3 months) [16], but also in guiding treatment decisions. It provides a means to assess pulmonary vasoreactivity and conduct hemodynamic evaluations, including identifying left-to-right shunts and addressing postcapillary confounders such as left ventricular disease and pulmonary vein stenosis. These factors are significant because they may either prohibit or limit the administration of pulmonary vasodilators. Further, some of these concomitantly detectable conditions (such as pulmonary vein stenosis) may warrant intervention. Despite its importance, the widespread acceptance and utilization of catheterization as a first-line diagnostic tool for BPD-PH face numerous challenges. These challenges include the availability of specialized teams comfortable with performing the procedure, such as cardiac interventionalists and cardiac anesthesiologists, along with considerations of patient risks. The BPD infant is particularly more susceptible to adverse effects from general anesthesia, reintubation, transport, and changes in temperature, which can result in clinical instability or even a PH crisis. Given these complexities, the current recommendation is to reserve cardiac catheterization for situations in which there are unexpected responses to initial PH-targeted therapy or the presence of unexplained, recurrent pulmonary edema. Further, cardiac catheterization should be considered when there is a suspicion of an anatomic abnormality; pulmonary vein stenosis on echocardiography or other cardiac imaging (magnetic resonance imaging (MRI) or computed tomography (CT) scan); aortopulmonary collaterals; congenital systemic-to-pulmonary shunt (PDA, ASD, or VSD); or when there is a concern for left ventricular functional alterations suggested by pulmonary edema, left ventricular hypertrophy, or left ventricular diastolic dysfunction on imaging.

### 2.4. Adjunct Testing May Be Helpful 

Laboratory testing may be performed as an adjunct to an echocardiographic screen or as follow-up investigations to monitor progression. Testing may include biomarkers such as baseline brain natriuretic peptide (BNP) or N-terminal pro-BNP (NT-proBNP) [62]. These peptides are cleaved fragments of pro B-type natriuretic protein (pro-BNP), a protein secreted by cardiomyocytes in response to stretch. Commercial and hospital-based laboratories can readily measure serum concentrations of these peptides, and they may be useful for trending over time. However, it is essential not to solely rely on them for making a diagnosis, because measured levels can be influenced by factors such as age (both gestational and postnatal), fluid status, systemic hypertension, and shunt volumes [61]. There is also an indication that pro-BNP values may be influenced by other complications of prematurity, including retinopathy of prematurity, sepsis, necrotizing enteropathy, and intraventricular hemorrhage [63]. BNP has a half-life of approximately 20 min, and NT-proBNP has a half-life of 2 h; therefore, both tests are limited in their ability to reflect the steady state [64]. Other commonly employed investigations in the BPD-PH population include serial electrocardiograms (with at least one upon suspicion of PH on echocardiography screening), serial blood gas analysis (given the direct correlation between respiratory acidosis and the deterioration of pulmonary vascular resistance), and serial expanded electrolytes (inclusive of calcium levels), especially in those exposed to chronic diuretics therapy [65]. Unfortunately, testing is often conducted using samples from acute venipuncture or capillary heel stick sources and, as a result, may not accurately reflect a true steady state. Additional testing includes imaging such as chest radiography, videofluoroscopic swallowing evaluation or flexible endoscopic evaluation of swallowing, laryngoscopy and bronchoscopy assessments, and oximetric evaluations, as well as computed tomography or magnetic resonance imaging of the chest to assess for other comorbidities that may cause or contribute to BPD-PH. The option for these studies may vary across institutions, reflecting the availability of testing and/or the availability of trained personnel to perform and interpret them. In certain clinical scenarios, an ACTH stimulation test for adrenal insufficiency, a thyroid function test, genetic evaluations for PH-specific mutations (such as TBX4 and BMPR2), and evaluations for confounding developmental lung disease conditions (such as errors of surfactant metabolism, alveolar capillary dysplasia, and filamin A) may be warranted. 

## 3. Challenge #2: Multitiered Management of BPD-PH

### 3.1. Focusing on Optimization of Respiratory Disease as First-Line Therapy 

Once BPD-PH is suspected and confirmed, either by serial echocardiography or cardiac catheterization, management is needed to prevent further morbidity and to minimize mortality. However, this poses a challenge for most clinicians because there are few medical therapies approved and studied in this population, and these infants have many confounding variables that can affect stability for therapeutic interventions. However, since the objective of BPD-PH management is to prevent progression of pulmonary vascular remodeling, the mainstays of treatment are optimization of lung growth, prevention of lung injury, and avoidance of a local hypoxic microenvironment [66]. This consists of judicious use of oxygen, positive airway pressure, and diuretics [67] to optimize pulmonary mechanics and acid-base status. 

Guidelines recommend initiating the management of BPD-PH by adopting a proactive approach to address BPD comprehensively. This involves conducting a thorough assessment to identify potential confounding variables that may impact the management of BPD-PH. These variables include conditions such as severe parenchymal lung disease, chronic reflux or aspiration, as well as airway abnormalities such as malacia [5,6,21]. Each of these conditions may contribute to BPD-PH and must be addressed while the infant is assessed for initiation of medical treatment with targeted PH therapies. Supporting lung growth to achieve adequate growth velocity (weight-for-length or body mass index), implementation of safe feeding practices with the goal of preventing aspiration, and maintaining adequate lung inflation will promote the growth of respiratory bronchioles and associated vasculature. Continued care in the outpatient setting to ensure vaccination, thoughtful timing for elective surgeries, and parental education is an additional cornerstone of treatment. This, which is the standard of care for neonatology, is the basis for BPD-PH management. Within these recommendations, however, there are nuances that are of particular importance for the BPD-PH infant and require specific PH expertise. 

### 3.2. Management of Chronic Respiratory Failure: Ventilator

Adequate management of chronic respiratory failure in some BPD-PH patients may completely alleviate PH altogether by minimizing local hypoxic, hypercapnic, compression, and distention effects on the pulmonary vasculature. Therefore, focusing on respiratory management is recommended as the first-line therapy. In those infants still on mechanical ventilation, utilizing a ventilator strategy to minimize hypercapnia and maintaining the infant as close as possible to functional residual capacity (FRC) using positive end-expiratory pressure (PEEP) (to overcome intrinsic PEEP for adequate inhalation and exhalation), adequate tidal volumes, and sufficient exhalation times can decrease additional strain on an otherwise already abnormal cardiorespiratory system. Ventilatory strategies for BPD recommend respiratory rates of 12–15 breaths per minute, tidal volumes of 10–12 millimeters per kilogram of body weight, and PEEP around 8–10 centimeters of water, although some infants with particularly severe BPD and large airway malacia may need higher PEEP levels to prevent air trapping and hyperinflation [21]. Using positive airway pressure to prevent alveolar derecruitment and ventilation–perfusion mismatch–related hypoxemia is a management tool for the BPD-PH population that cannot be overstated. Often, the timing for shifting the focus from BPD prevention strategies such as low PEEP, a higher respiratory rate, and a low inspiratory time to the management of established BPD to provide sufficient ventilatory support to facilitate growth and development is unclear and often needs a continuous assessment of the patient through a dedicated team with a multidisciplinary approach [9,61,68]. In some instances, tracheostomy may be needed to adequately meet the infant’s respiratory needs, while still allowing for adequate participation in therapies and promoting interaction and neurodevelopment of the infant at a critical stage of maturation. In addition to the use of positive airway pressure therapy, management of chronic respiratory failure also requires assessment of the airway to address any underlying tracheomalacia and/or bronchomalacia. 

### 3.3. Management of Chronic Respiratory Failure: Oxygen

Oxygen should be utilized to maintain oxygen saturations in a target range that will minimize hypoxic vasoconstriction. Saturation targets and ranges may vary based on the infant’s underlying disease driver and condition. However, because most BPD-PH infants are diagnosed after 36 weeks PMA, the use of oxygen should be less of a concern with relation to retinopathy of prematurity. For the majority of term, uncomplicated BPD-PH infants (i.e., without pulmonary vein stenosis or cardiac shunts), targeting saturations of 92–95% is reasonable [5,61,69]. Some guidelines have advocated for the use of 95% saturation as a target in infants with established BPD-PH to avoid hypoxemic stress on the pulmonary vasculature, although no trial has tested this saturation target within this population [70,71]. Nevertheless, when evidence of PH is present on echocardiography before 36 weeks of PMA, there is a lack of data regarding the target oxygen saturations and their association with long-term outcomes. Addressing this gap requires a multicenter approach to study these effects over the long term.

### 3.4. Identifying Confounding Variables: Left-to-Right Intracardiac Shunts, Pulmonary Vein Stenosis

Management of the BPD-PH infant must include assessment of left-to-right intracardiac and/or extracardiac shunts, such as atrial septal defects and PDA. While these have been considered benign in the general pediatric population, there is an increased sensitivity to recognizing the negative effects that additional pulmonary blood flow (and pressure transmission) can have in some BPD infants, potentially even contributing to the development of BPD [72,73]. While not directly proportional, it is observed that the younger the gestational age at birth, the more simplified the pulmonary parenchyma is, and the more marked the premature postnatal disruption in respiratory and vascular development [74], which may be observed as a diminished tolerance of increased pulmonary blood flow from shunt sources. Therefore, in situations in which the infant is unable to make clinical advancements, unable to wean from respiratory support or diuretics, and exhibits poor growth and poor tolerance of handling, consideration for closure of the left-to-right shunt could be considered [68,72,73]. Multidisciplinary discussion is recommended to evaluate risks, benefits, and timing of this intervention [68]. However, the presence of an interatrial shunt or post-tricuspid shunt, in the context of PH, may also be kept open in order to allow a pop-off in cases of PH crisis. As such, each case of suspected pulmonary vascular disease should be thoroughly discussed and evaluated in a multidisciplinary fashion to evaluate if the flow lesion contributes to the overall increase in pulmonary arterial pressure, or if the predominant phenotype is one of underlying high pulmonary vascular resistance.

Echocardiography can offer valuable insights into pulmonary vein stenosis, indicated by elevated velocity (a mean gradient exceeding 4 mmHg) and the loss of phasic flow [3]. However, it is important to note that echocardiography might not detect multilevel stenosis or pulmonary venous atresia, and the visibility of pulmonary veins can be compromised depending on the acoustic windows availability. Therefore, computed tomography of the chest is recommended for optimal visualization of the pulmonary veins [3,51]. Infants with pulmonary vein stenosis may often present with recurrent or persistent pulmonary edema, tachypnea/respiratory distress, or poor tolerance of fluids.

### 3.5. Treating Comorbidities to Minimize Respiratory Complications

#### Nutrition and Feeding 

Nutrition and management of fluids is an extremely important treatment for BPD-PH infants to support lung growth and minimize fluid overload into leaky pulmonary capillaries of preterm infants. Nutritional support may be complicated by fluid restriction and/or the use of diuretics. However, caloric fortification should be offered when appropriate and possible with close monitoring of weight gain, linear growth, and head circumference [75,76,77]. Collaboration with dietitians well versed in neonatal nutrition should be sought and tracking of weight/length ratio and body mass index are important as part of the global monitoring of well-being [78]. 

Gastroesophageal (GE) reflux is common in preterm infants and especially in those with BPD, with up to 40% of reported prevalence [79]. The diagnosis and management of GE reflux microaspiration in the NICU setting is challenging due to variable practices across institutions. Although the correlation between GE reflux symptoms and the severity of lung disease is not direct, most PH guidelines recommend addressing GE reflux in infants with severe BPD to minimize PH-related events [80,81,82]. Dynamic airway collapse in addition to GE reflux and laryngeal injury probably predisposes to an increased aspiration risk in preterm infants with BPD [83]. Recurrent small amounts of aspiration can be silent or may present with nonspecific clinical symptoms, such as an increased work of breathing, wheezing, a frequent drop in oxygen saturations, or poor weight gain, and can be challenging to diagnose. Tests such as the Flexible Endoscopic Evaluation of Swallowing and the barium swallow study lack sensitivity for aspiration risk in preterm infants. Therefore, the active participation of a dedicated team of speech and occupational therapists in their ongoing evaluation, as well as for the bedside assessments, is crucial. Promoting safe feeding practices to minimize aspiration is critical to lung health and continued healthy lung function [1,75,78,84]. 

### 3.6. Fluid Management

Infants with BPD-PH are particularly sensitive to alterations in fluid status. An increased pulmonary blood flow relative to the parenchymal development or poor fluid clearance from immature or injured kidneys can result in a suboptimal fluid balance and cause additional cardiorespiratory strain [84]. Diuretics are therefore a mainstay of treatment in this population, often used to reduced pulmonary vascular congestion. With oxygen, diuretics are among the most widely prescribed therapy for BPD-PH infants [85,86]. Ongoing monitoring of chemistry and growth is critical while on diuretics or fluid restriction. Fluid status must be assessed regularly to take care to avoid low preload and cardiac output conditions. Additionally, observational data suggest that diuretic therapy may be associated with improvement in pulmonary pressures and right and left ventricular function in BPD-PH infants [67]. Future studies should also evaluate the contribution of altered LV diastolic performance and increased systemic vascular tone as potential contributors to the BPD-PH phenotype [33,87]. Indeed, new therapeutic avenues to be studied may involve LV afterload reduction in such circumstances.

### 3.7. Lack of Approved PH Therapies and Lack of Data in This Population Should Not Deter Use When Indicated

#### PH Pharmacotherapy

When optimization of ventilation, oxygenation, nutrition, fluid balance, acid-base balance, infectious status, and any intracardiac shunts have failed to improve clinical status or indices of PH in the BPD-PH infant by echocardiogram, lab monitoring, or cardiac catheterization, PH-specific pharmacotherapy should be considered as the next line in management. This may present as the infant who, despite optimized gas exchange, appropriate lung expansion on chest imaging, minimization of aspiration and malacia, and judicious fluid management, continues to exhibit poor growth, intolerance of cares, and a persistently abnormal echocardiogram. There are multiple classes of therapy that may be utilized with the goal of promoting pulmonary vasodilation and preventing vascular remodeling that may lead to irreversible vasoconstriction and cardiopulmonary morbidity. Three broad classes of PH-specific vasodilators are typically utilized by PH specialists in the management of BPD-PH, targeting the nitric oxide pathway, endothelin pathway, and prostacyclin pathway. While none of these agents have been extensively studied in controlled clinical trials in the population affected with BPD-PH [88], there are numerous cohort studies, case reports, and reviews that attest to their safety, tolerability, and efficacy using standard PH dosing regimens in appropriately selected patients. Newer agents such as those acting on the soluble guanylate activation (i.e., riociguat) or oral prostanoids activators (i.e., selexipag) have limited data within the BPD-PH population. The arsenal of PH-specific vasodilators continues to grow but familiarity with the various classes, formulations of therapies, and specific use in the BPD-PH population is limited to PH specialists who may not be readily available for consultation in BPD-PH infant care. Despite this lack of universal experience, consultation with a specialist experienced in the use of these medications is recommended prior to initiation of therapy. 

Nitric oxide pathway: The AHA/ATS PH guidelines [5] recommend the use of inhaled nitric oxide (iNO) for pediatric cases experiencing a PH crisis. iNO can be delivered through invasive or noninvasive respiratory support and has the potential to rapidly induce pulmonary vasodilation and improve ventilation–perfusion mismatch and therefore rapidly drop the RV afterload. As such, treatment with iNO has been advocated for infants diagnosed with BPD and symptomatic PH [21]. iNO binds to soluble guanylate cyclase in the pulmonary vascular endothelial cells, stimulating the conversion of guanosine triphosphate to cyclic guanosine monophosphate (cGMP), which, as a second messenger, prompts the relaxation of smooth muscle cells in blood vessel walls. Exogenous iNO is often used in infants with other forms of pulmonary arterial hypertension, particularly in the setting of persistent pulmonary hypertension of the newborn (PPHN) [18,69]. Given the effect of nitric oxide in vasodilation, iNO is often used beyond the perinatal period as an acute, temporary therapy for the BPD-PH infant. This may be considered episodically for severe hypoxemia or to facilitate medication adjustment. 

Phosphodiesterase-5 inhibitors (PDE-5i) also increase intracellular cGMP levels, promoting vasodilation in the nitric oxide pathway. There are animal reports that nitric oxide may promote continued lung development as well, although this has not been readily demonstrated or studied in the developing human lung [89]. Sildenafil is currently approved by the Food and Drug Administration (FDA) in children greater than one year of age. It remains the most widely used PH therapy for BPD-PH [90,91,92] and study as a possible preventative therapy is being explored [93,94]. Adverse drug effects of GE reflux [95] are not uncommon in the BPD-PH population and infants should be monitored for this. Tadalafil use has increased for BPD-PH therapy owing to the perceived lack of GE reflux exacerbation, although this is largely anecdotal. PDE-5i administration should be monitored for hypotension. Riociguat, a soluble guanylate cyclase stimulator, enhances the generation of endogenous nitric oxide. While approved for World Symposium of Pulmonary Hypertension (WSPH) Group 1 and Group 4, it is not sanctioned for Group 3, which encompasses BPD-PH. Nonetheless, anecdotal evidence and animal studies hint at its potential efficacy in Group 3 infants, indicating a prospective role in future BPD-PH therapy [96,97].

Acting via a similar pathway, milrinone is a phosphodiesterase-3 (PDE-3) inhibitor, leading to increased cAMP within cardiac and vascular smooth muscle cells. This results in enhanced cardiac contractility, filling, and relaxation of smooth muscle in blood vessels, leading to systemic and pulmonary vasodilation. While there is a scarcity of data specifically addressing BPD-PH, milrinone could be considered for those with suprasystemic PH and/or concomitant RV dysfunction. However, milrinone requires intravenous administration, may accumulate in renal impairment, and could precipitate systemic hypotension. It is important to recognize that all systemically administered pulmonary vasodilators have the potential to indiscriminately dilate pulmonary vessels. This could exacerbate ventilation–perfusion mismatch and lead to systemic hypoxia, particularly in infants with severe concurrent pulmonary parenchymal disease.

Endothelin pathway: Endothelin receptor antagonists (ERAs) block the endothelin receptor, preventing endothelin from binding to cause vasoconstriction and proliferation. Bosentan, an endothelin receptor A and receptor B antagonist, is metabolized by the liver and therefore can interact with many medications. Monthly monitoring, at a minimum, is required to ensure that liver function is not affected by its use. Bosentan is currently approved by the FDA for use in pediatric PH in children over three years of age, but is commonly utilized in the BPD-PH population. The other ERAs on the market (ambrisentan, macitentan) have limited reports in this population. They are attractive options because they have less effect on liver function and are selective antagonists, but may be associated with anemia and fluid retention. 

Prostacyclin: Prostacyclin’s effect is mediated through the activation of cyclic adenosine monophosphate (cAMP), which inhibits calcium influx into smooth muscle cells, leading to relaxation and dilation of blood vessels. This class of medications can be dosed by inhalation, oral, or continuous infusion (subcutaneous or intravenous). Side effects include flushing, systemic hypotension, pulmonary edema and pleural effusions, local skin reaction, tachycardia, fever, worsening of ventilation–perfusion mismatch, and more. These limitations frequently restrict their application in more delicate BPD-PH infants, leading to their reserved usage primarily in severe PH cases. Prostacyclin may be used acutely in the immediate management of RV failure or PH crisis or long term with noted improvement. 

## 4. Challenge #3: Long-Term Follow-Up Care of BPD-PH Infants

The long-term follow-up care of the BPD-PH infant demands a multidisciplinary team approach to ensure that all aspects of treatment listed above are addressed [68]. This is crucial given the high risk of mortality, a risk that increases with the degree of PH [9,98]. Compared to BPD infants without PH, BPD-PH infants have higher rates of needing technology including respiratory support, feeding support, and oxygen therapy and readmissions with illness are not infrequent [7]. With expectant management, avoidance of illness, and good growth, BPD-PH is generally felt to improve over the course of the first few years of life, allowing for the discontinuation of PH therapies by a median of 4.4 years [88,99]. However, the postdischarge course of a BPD-PH infant requires close follow-up because negative cardiorespiratory outcomes can persist well beyond infancy and even into adulthood [7,100,101].

## 5. Overcoming the Challenges Listed 

**Figure 1:**
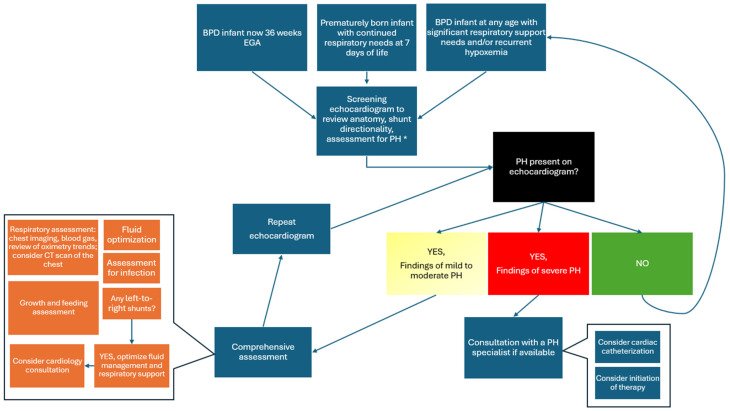
Proposed algorithmic approach to BPD-PH. * Consideration should be given for robust PH assessment using echocardiography metrics noted in Table 1.

An unexpected hurdle in implementing screening guidelines lies in determining the appropriate course of action based on the results. If a BPD infant presents with an abnormal echocardiogram, the consensus is that a comprehensive assessment (Figure 1), with particular attention to respiratory status, should take precedence as the first line of management. Prior to initiating any medication therapy, it is crucial to prioritize the optimization of respiratory, fluid, or infectious status (with appropriate protective measures such as infant and family vaccination), because changes observed on echocardiography may potentially improve or resolve through these measures. After optimizing the infant to the best of the bedside clinician’s ability, the subsequent steps may involve confirming the BPD-PH diagnosis through cardiac catheterization or further imaging (MRI or CT scan) to assess for parenchymal disease or pulmonary vein stenosis. However, since cardiac catheterization may not be readily available in all institutions, it is recommended to conduct hemodynamic catheterizations in expert centers to minimize complications [102]. Moving to a facility with this expertise may not always be feasible, prompting clinicians to consider treatment with PH vasodilators for early stabilization and escalation with continued clinical distress. A challenge here lies in the limited availability of robust data, which may cause hesitation among less experienced clinicians or result in inappropriate medication choices or regimens. In clinical practice, it is not uncommon for high doses of sildenafil and bosentan to be employed as a “last resort,” but the authors caution against that because it can potentially result in an unsafe situation with a risk of iatrogenic patient harm. 

Each neonatology practice will encounter unique considerations in treating BPD-PH, impacting available treatment options. Limitations may differ based on institution and country, including formulary restrictions, local regulations, drug availability, market access, delivery methods, national regulations by drug agencies (i.e., “risk evaluation and mitigation strategy” programs), limited hospital formulary access, logistical challenges in medication administration, and insufficient expertise or guidance from pulmonary hypertension consultants. Additionally, familiarity with optimal dosing regimens can also influence treatment strategies. Whenever possible, consultation with an experienced BPD-PH specialist should be sought, either within the institution or by external consultation. While selecting the optimal vasodilator of the BPD-PH infant, thought must be given to each individual patient’s physiology and patient-specific factors such as vasoreactivity on cardiac catheterization, existing left-to-right intracardiac shunts, left heart disease, and pulmonary vein stenosis, amongst others. These factors may not only guide which agent may be best tolerated, but also which may be the most efficacious. It is critical to note that systemically absorbed pulmonary vasodilators may exert an undifferentiated effect on the pulmonary vasculature, potentially contributing to ventilation–perfusion mismatch. Therefore, once PH-specific pharmacotherapy is initiated, close monitoring of clinical parameters, echocardiogram or catheterization indices, and laboratory monitoring is recommended to ensure tolerance and monitor for efficacy. 

Overcoming these numerous challenges is, unfortunately, not a straightforward task. Addressing these issues requires an understanding of the local culture, availability of experienced consultants, and proximity to larger comprehensive centers. Therefore, there is no one-size-fits-all solution that can be incorporated into guidelines to overcome these barriers to implementation. It should be acknowledged that although PH expertise may not be readily available in the original institution, consultation via phone or through a formal second opinion may be possible. If the infant is safe for transport, consideration may be given to transferring them to an institution with BPD-PH expertise for acute stabilization, confirmation of diagnosis, and initiation of treatment.

## 6. Conclusions

The development of BPD-PH unfortunately represents a significant and serious complication for infants with BPD. Due to the premature disruption of respiratory and vascular development, the risk for postnatal complications is notably high. Guidelines are available for screening and diagnosing PH in this population, and there is an increasing body of experience with PH treatment, particularly concerning supportive care such as managing chronic respiratory failure, providing nutritional support, and intervening on intracardiac shunts. However, there are multiple practical challenges at the bedside that may impact the implementation and, ultimately, the management of this life-threatening complication. While general recommendations exist to address these barriers, further studies are needed to incorporate them into subsequent practice models.

## Figures and Tables

**Table 1:** Echocardiography metrics for BPD-PH with references.

Estimation of Pulmonary Pressures	
RVSP estimation by tricuspid regurgitant jet velocity	Concern of PH if the RVSP > 40 mmHg by the TR jet (TR jet with an RV–RA gradient of >35 mmHg, assuming an RA pressure of 5 mmHg). RVSP > 1/2 of systemic pressure is concerning for abnormal pulmonary pressure. About 60% of echocardiograms may have a quantifiable TR jet with a full envelope. Some use the cutoff of the TR jet > 33.6 mmHg) [12,22].
Mean and diastolic PAP by pulmonary insufficiency jet	Often not available in BPD-PH scans. However, when available, it provides estimates of PAP during the diastolic phase of the cardiac cycle [24,25]. (mPAP = 4 × [early diastolic PI velocity]2 + estimated RA pressure)
Gradient and directionality through restrictive PDA or VSD	May inform on systemic-to-pulmonary relationship based on directionality. Velocity gradient may inform on sPAP and dPAP when compared to systemic blood pressure at the time of the echocardiogram. Equalization of pressures occurs with unrestrictive shunts—limiting the interpretation of underlying PVR [12,24].
Pulmonary artery acceleration time/right ventricular ejection time (PAAT/RVET)	This ratio provides some insight on the RV afterload. In a situation where the RV afterload is increased (either due to high pulmonary vascular resistance or other contributors—flow/pressure transmission), this ratio decreases. Ratio is measured from the pulsed wave (PW) Doppler envelope of the right ventricular outflow tract. A low ratio suggests an increased pulmonary afterload (abnormal < 0.31; some use a cutoff of <0.25) [26,27,28].
LV eccentricity index at peak systole	The left ventricular (LV) end-systolic eccentricity index provides a quantifiable metric of septal deformation. The index is computed as the ratio of the diameter parallel to the septum to the diameter perpendicular to the septum at peak of systole. In a situation where there is a flat septal configuration or a bowing septum, this ratio will decrease. This provides a continuous quantifiable metric of the “septal motion.” In the absence of a congenital cardiac anomaly, ventricles will equalize pressure with their corresponding outflow tract at the peak of systole. As such, the RV–LV relationship may inform on the systemic-to-pulmonary systolic pressure relationship. In the expected setting, the LV systolic pressure should be above the RV systolic pressure, and the LV should form a near-perfect circular configuration at the peak of systole. The left ventricular (LV) end-systolic eccentricity index (EI) ≥ 1.3 has been associated with PH in BPD infants [29,30].
LV septal motion	Septal flattening (or bowing toward LV) at peak of systole indicates an increased RV–LV systolic pressure relationship. Flattening concerning for systolic PA pressure is greater than 50% systemic pressure [31].
**Evaluation of RV Function/Dimensions**	
TAPSE	Tricuspid annular plane systolic excursion (TAPSE) is a marker evaluating RV systolic function using the M-Mode tracking motion of the tricuspid valve (line of interrogation crossing the apex and attachment of the tricuspid valve to the RV-free wall). It estimates the longitudinal displacement of the tricuspid valve from peak diastole to peak systole. Low values (by age) indicate RV dysfunction [32,33,34].
RV-FAC	FAC is calculated after obtaining the end diastolic (EDA) and the end systolic area (ESA) of the RV (FAC = [EDA − ESA]/EDA), and also provides an important marker of RV function. Normative values have been published (although normal FAC values quoted to be most common when >35%) [35,36,37].
RV-MPI by TDI	Evaluates the RV myocardial performance index using tissue Doppler imaging. Combined marker of RV systolic and diastolic performance [35].
RV output estimation	Assesses RV stroke volume and cardiac output. Values < 150 mL/kg/min are of concern [26,38].
RV E/A ratio	Assesses RV diastolic function [37].
RV S’ by TDI	Measures RV systolic velocity using tissue Doppler imaging (peak longitudinal contraction velocity). May be decreased in the context of systolic dysfunction [37,39,40].
RV E/E’ by TDI	Estimates RV filling/diastolic function [37,39,40].
RV EDA	Evaluates RV end-diastolic area [37].
RV/LV	RV/LV ratio > 1 at peak of systole in parasternal short axis is concerning for RV dilation [24,41].
RV longitudinal strain	Speckle-tracking echocardiography allows for assessment of RV longitudinal deformation during contraction. Associated with later mortality in those with BPD-PH diagnosis. Normative values have been published by age and vendor [32,33,42,43].
**Evaluation of LV Function/Dimensions**	
Shortening fraction	May be computed from the 2D or Motion-Modes. Ratio between the end diastolic and peak systolic diameters of the internal cavity of the LV at the tip of the mitral valve. Concern with angle of image acquisition and assessment of partial/segmental LV function. Normal: 28–46% [44].
EF by biplane	Standard measure of LV ejection fraction. Assumes a mathematical bullet-shaped LV, which may not be true in the context of adverse septal motion. Normal > 55% [44,45].
EF by 5/6 area length	Alternative measure of LV ejection fraction. Normal > 55% [46].
LV-EDV	LV end-diastolic volume assessment [44,46].
LV mass	Assesses LV hypertrophy, potentially leading to a decreased LV compliance and an increased end-diastolic pressure [47].
LV output estimate	Evaluates LV stroke volume and cardiac output. Values < 150 mL/kg/min are of concern [26].
LV S’ by TDI	Measures LV systolic velocity using tissue Doppler imaging (peak longitudinal contraction velocity). May be decreased in the context of systolic dysfunction [39].
LV E/E’ (free wall and septal)	Estimates LV filling pressures [39].
LV E/A	Assesses LV diastolic function [48].
LV longitudinal or circumferential strain	Speckle-tracking echocardiography allows for assessment of LV longitudinal and circumferential deformation during contraction. In healthy children, the mean LV global longitudinal strain is −20% (95% CI, −19.5% to −21%) and the mean global circumferential strain is −22% (95% CI, −20% to −25%) [33,42,43].
**Evaluation of Shunts**	
Atrial shunt evaluation	A bidirectional or right-to-left shunt suggests higher right-sided atrial pressure (often secondary to RV diastolic dysfunction). These patients may have concomitant hepatomegaly and dilated inferior vena cava and subhepatic veins (with occasionally >50% retrograde flow by pulse-wave Doppler) [26,28,49].
Post-tricuspid shunt evaluation	A bidirectional or right-to-left shunt provides information on the pressure relationship between the pulmonary and systemic sides; unrestrictive shunts lead to equalized systolic pressure [26,28,49].
**Assessment for Concomitant Anomalies**	
Pulmonary veins	Assessment of pulmonary venous flow at each ostium to rule out signs of pulmonary veins stenosis (mean gradient < 4 mmHg, biphasic/triphasic flow) [50,51].
Pulmonary valve, main pulmonary artery and right/left pulmonary arteries	Evaluation for signs of RV outflow tract obstruction, or stenosis/obstruction in the pulmonary arteries [10].
**Future Parameters in Investigation**	
LV-EF by 3D echocardiography	3D-volume capture of the LV in order to estimate LV dimensions and function by ejection fraction (or strain). Limited data are available in the neonatal population [52].
RV-EF by 3D echocardiography	Detailed RV ejection fraction assessment using 3D-echocardiography and modeling. Advanced techniques are often challenging in preterm infants and require specific expertise, equipment, and assessment tools [53].
Blood speckle-tracking	Assessment of vortex formation in the LV and RV. Estimation of transcavitary pressure gradients [54].
Myocardial work assessment	Incorporates markers of left or right ventricular afterload into strain analysis [55].

Table provided by Dr. Gabriel Altit.

**Table 2:** Selected image examples of metrics used in the assessment of BPD-PH.

Metric	Echocardiography Image Example
Pulmonary insufficiency jet	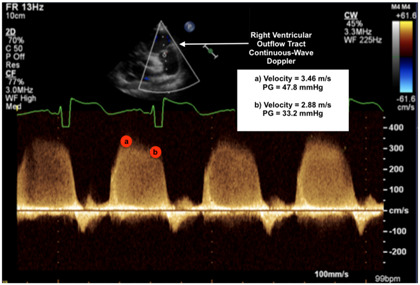
	Legend: Mean pulmonary arterial pressure (mPAP): Peak/early diastolic velocity estimates the PA–RV (pulmonary artery to right ventricle) gradient in early diastole to be 32.59 mmHg using the modified Bernoulli equation (4 × velocity^2^). As such, mPAP is estimated as 47.8 mmHg (4 × 3.46^2^) + expected right atrial (RA) pressure (~5 mmHg) = 52.8 mmHg [24].
	Diastolic pulmonary arterial pressure (dPAP): End-diastolic velocity estimates the PA–RV gradient in late diastole to be 33.2 mmHg using the modified Bernoulli equation. As such, dPAP is estimated as 33.2 mmHg + expected RA pressure = 38.2 mmHg [24].
TAPSE—tricuspid annular plane systolic execution	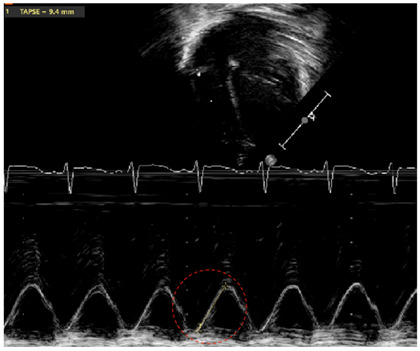
	Legend: Marker of longitudinal systolic function of the right ventricle (RV) (which primarily contracts longitudinally). The M(motion)-Mode is used with the line of interrogation passing through the attachment of the tricuspid valve at the level of the free wall of the RV, and through the RV apex. The distance travelled from end-diastole to peak of systole is measured by following the line of the attachment of the tricuspid valve on the M-Mode tracing through time (red circle). Occasionally, superimposed tissue Doppler allows to follow the period of systole with increased precision. Z-scores have been published by gestational age/postmenstrual age, as well as chronological age for term infants [34,56].
Tricuspid regurgitation jet	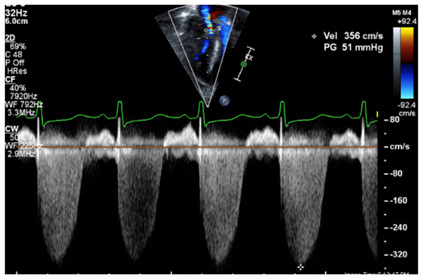
	Legend: The tricuspid regurgitant jet velocity provides information on the velocity of flow during systole from the RV to the RA. This allows to estimate, using the Bernoulli equation, the pressure gradient between the RV and the RA. A full envelope is generated when the line of interrogation is parallel to the tricuspid regurgitant jet. With an estimated RA pressure (typically 0–5 mmHg in a normal setting, but likely further increased in RV diastolic impairment), one is able to estimate the peak systolic RV pressure. Assuming that the RV and pulmonary arterial pressure are equalized at the peak of systole, one may infer the peak systolic pulmonary arterial pressure. The velocity measurement should be performed along the contour of the spectral Doppler envelope, avoiding overestimation of the measurement. In this case, the peak systolic RV–RA gradient has a velocity of 3.56 m/s, providing an estimated RV–RA pressure gradient of 51 mmHg (4 × 3.56^2^). With an RA pressure of 5 mmHg, this provides a peak systolic RV pressure estimated at 56 mmHg (abnormal > 40 mmHg).
Fractional area of change by RV	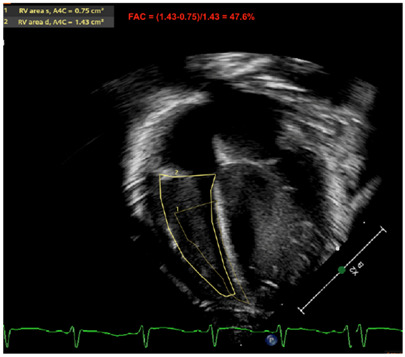
	Legend: The fractional area change of the RV is another marker used to estimate RV systolic function. The contour at the endocardial border is traced at the peak of systole and at the peak of diastole. The fraction ([RV End Diastolic Area] − [RV End Systolic Area])/[RV End Diastolic Area] is expressed in % of area shortening. An apical RV focused view is used. This marker may be calculated in the apical four-chamber view. Some reports have also used the RV inflow–outflow view (or RV three-chamber view, or RV “tet” view—which outlines the RV anterior and inferior walls) to compute this metric, although the American Society of Echocardiography officially recommends its evaluation in apical four-chamber view—which outlines the RV free wall and RV septum. Here, the RV-FAC is 47.6%, which is considered normal (>35%)
RV E/A ratio	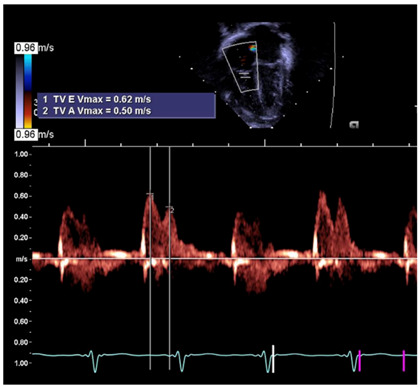
	Legend: E (early filling velocity), A (late/atrial contraction filling velocity). This metric is used in the pediatric literature to evaluate diastolic performance. Data are lacking regarding the newborn. However, under normal circumstances at a few weeks of life, it is expected that the RV compliance is now increased (compared to the early postnatal period) and that the filling in the early phase (passive) occurs at a higher velocity than during the atrial contraction. Here, the ratio is >1.0, indicating that the E>A (0.62 m/s > 0.50 m/s)—which is considered normal.
PAAT/RVET	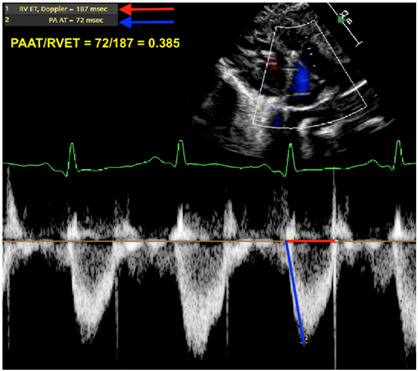
	Legend: The pulmonary artery acceleration time to the right ventricular ejection time is a ratio providing some insight on the RV afterload. In situations where the RV afterload is increased (either due to high pulmonary vascular resistance or other contributors—flow/pressure transmission), this ratio decreases. The pulsewave Doppler envelope of the RV outflow tract shifts from a parabolic shape to a more triangular shape with a steeper diastolic upstroke. Here, the ratio is 0.385 (abnormal < 0.31; some use a cutoff of <0.25).
Eccentricity index	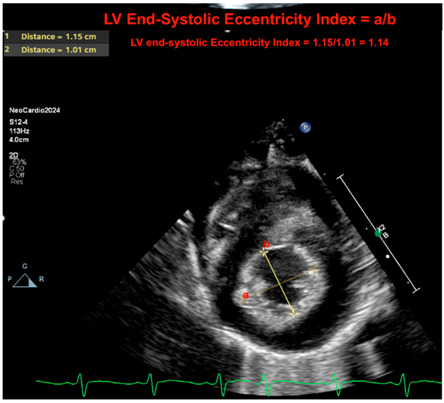
	Legend: The LV end-systolic eccentricity index provides a quantifiable metric of septal deformation. The index is computed as the ratio of the diameter parallel to the septum to the diameter perpendicular to the septum at peak of systole. In situations where there is a flat septal configuration or a bowing septum, this ratio will decrease. This provides a continuous quantifiable metric of the “septal motion.” In the absence of a congenital cardiac anomaly, ventricles will equalize pressure with their corresponding outflow tract at the peak of systole. As such, the RV–LV relationship may inform on the systemic to pulmonary systolic pressure relationship. In the expected setting, the LV systolic pressure should be below the RV systolic pressure, and the LV should form a near-perfect circular configuration at the peak of systole. Here, the ratio is 1.14 (normal if <1.3). Letters a = 1; b = 2. The equation is a/b.
